# Reduced Contact Resistance Between Metal and n-Ge by Insertion of ZnO with Argon Plasma Treatment

**DOI:** 10.1186/s11671-018-2650-y

**Published:** 2018-08-15

**Authors:** Yi Zhang, Genquan Han, Hao Wu, Xiao Wang, Yan Liu, Jincheng Zhang, Huan Liu, Haihua Zheng, Xue Chen, Chang Liu, Yue Hao

**Affiliations:** 1 State Key Discipline Laboratory of Wide Band Gap Semiconductor Technology, School of Microelectronics, Xidian University, Xi’an, 710071 People’s Republic of China; 2 Key Laboratory of Artificial Micro- and Nano-structures of Ministry of Education, School of Physics and Technology, Wuhan University, Wuhan, 430072 People’s Republic of China

**Keywords:** Germanium, Fermi-level pinning, Ohmic contact, Argon plasma, ZnO

## Abstract

We investigate the metal-insulator-semiconductor contacts on n-Ge utilizing a ZnO interfacial layer (IL) to overcome the Fermi-level pinning (FLP) effect at metal/Ge interface and reduce the barrier height for electrons. A small conduction band offset of 0.22 eV at the interface between ZnO and n-Ge is obtained, and the ZnO IL leads to the significant reduced contact resistance (*R*_c_) in metal/ZnO/n-Ge compared to the control device without ZnO, due to the elimination of FLP. It is observed that the argon (Ar) plasma treatment of ZnO can further improve the *R*_c_ characteristics in Al/ZnO/n-Ge device, which is due to that Ar plasma treatment increases the concentration of oxygen vacancy *V*_o_, acting as n-type dopants in ZnO. The ohmic contact is demonstrated in the Al/ZnO/n-Ge with a dopant concentration of 3 × 10^16^ cm^−3^ in Ge. On the heavily doped n^+^-Ge with a phosphor ion (P^+^) implantation, a specific contact resistivity of 2.86 × 10^− 5^ Ω cm^2^ is achieved in Al/ZnO/n^+^-Ge with Ar plasma treatment.

## Background

Germanium (Ge) has attracted much attention for the advanced metal-oxide-semiconductor field-effect transistors (MOSFETs) due to its higher carrier mobilities compared to Si [1, 2]. For the Ge p-channel MOSFETs, great progress has been made in growth of strained Ge channel [3–5], surface passivation [6–9], and source/drain (S/D) contacts [10], and the ultra-scaled Ge pFinFETs [11] have demonstrated the superior electrical performance to the Si devices. Ge n-channel transistors, by contrast, are still facing challenges, which produce the obstacle for the integration of Ge CMOS, including the poor interface quality, resulting in the low electron mobility, and the high S/D resistance due to the limited activation rate of n-type dopants in Ge [12] and the Fermi-level pinning (FLP) at metal/n-Ge interface [13]. FLP leads to a Schottky barrier height of about 0.5 eV for electrons for most of the metals on n-Ge, producing the very large contact resistance *R*_c_ [13–15].

Fermi-level depinning can be done by inserting a thin interfacial layer (IL), e.g., TiO_2_ [16] and ZnO [17], between the metals and n-Ge [18], due to that the thin IL can block the metal wave function into Ge to reduce the metal-induced gap states [19, 20]. ZnO has small conduction band offset (CBO) with respect to Ge, which can lead to the smaller *R*_c_ in metal/ZnO/n-Ge, compared to metal/TiO_2_/n-Ge with TiO_2_/Ge having the positive CBO [16]. The dielectric constant of ZnO is smaller than that of TiO_2_, so ZnO IL can obtain a thinner depletion region between the metal and n-Ge in comparison with TiO_2_. In addition, it is easy to realize n-type doping in ZnO by introducing nonstoichiometric defects, such as oxygen vacancies *V*_o_ [21, 22], which gives rise to an even smaller ZnO depletion region between the metal and n-Ge. So far, in metal/ZnO/n-Ge contacts, the doping of ZnO by *V*_o_ was carried out by annealing in nitrogen atmosphere [16], which however, might resulted to the inter diffusion of ZnO and Ge during the annealing [23], and diffusion of dopant atoms in n-Ge during the annealing [24, 25], causing the degradation of current performance of the device. Since, a low-temperature process for depositing and doping ZnO needs to be developed.

In this work, we investigate the Fermi-level depinning at interface between metal and n-Ge by insertion of ALD deposited ZnO IL. The improvement effects of argon (Ar) plasma treatment of ZnO layer on contact resistance characteristics of Al/ZnO/n-Ge are studied.

## Methods

Metal contacts were formed on both lightly and heavily doped n-Ge (001) wafers. The lightly doped Ge samples have a doping concentration about 3 × 10^16^ cm^−3^. To achieve the heavily doped n-Ge, a phosphor ion (P^+^) implant with a dose of 1 × 10^15^ cm^−2^ and an energy of 30 keV was performed on the n-Ge(001), which was followed by a rapid thermal annealing at 600 °C for 60 s. After wafer cleaning using several cycles of deionized water and diluted HCl, Ge wafers were immediately loaded into ALD (Beneq TSF-200) chamber to deposit ZnO, and then aluminum (Al) contacts were deposited by sputtering on Ge using a lift-off process. Here, three ZnO thicknesses of 1, 2, and 3 nm were utilized, which were confirmed by spectroscopic ellipsometry (SE) (J. A. Woollam M2000). During the ZnO deposition, diethyl zinc (DEZn) and deionized water (H_2_O) were used as the Zn and O precursors, respectively, and the substrate temperature was kept 150 °C to eliminate the formation of GeO_*x*_. The detailed ZnO deposition process using ALD was reported in our previous works in ref. [26, 27]. To further improve the conductivity of ZnO film, some ZnO on Ge samples were treated with argon (Ar) plasma. Control Al/n-Ge sample without ZnO IL was also fabricated. The *R*_c_ of Al on ZnO/Ge was extracted using the circular transmission line method (CTLM), which was formed by lift-off. The exposed ZnO was fully etched by plasma etch to ensure complete isolation between adjacent devices [16].

Keithley 4200 SCS was used to measure the electrical characteristics of the Al/ZnO/n-Ge contracts and CTLM structures, high-resolution transmission electron microscope (HRTEM) and X-ray photoelectron spectroscopy (XPS) were used to determine the microstructure and interface properties of the samples, and UV-VIS Spectrophotometer (LAMBDA 950, PerkinElmer) was used to determine the band gap *E*_g_ of deposited ZnO film.

## Results and Discussion

### Material Characterization of Al/ZnO/n-Ge

**Fig. 1:**
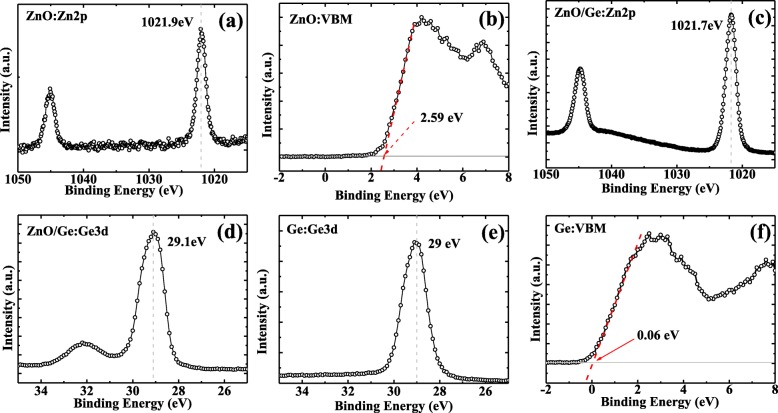
XPS spectra for valence bands of ZnO/Ge sample. **a** Zn 2*p* and **b** valence band spectra for thick ZnO sample **c** ZnO 2*p* and **d** Ge 3*d* spectra for ZnO/Ge interface sample, and **e** Ge 3*d* and **f** valence band spectra for bulk Ge sample

**Fig. 2:**
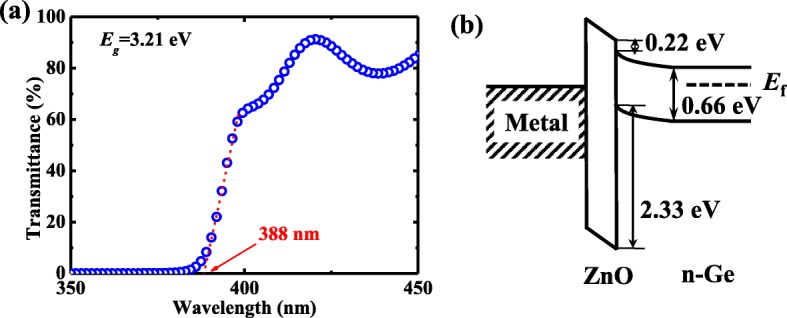
**a** Transmittance spectrum for the deposited ZnO film demonstrating the *E*_g_ of 3.21 eV. **b** The band alignment for Al/ZnO/Ge contact

XPS valence band spectra of Ge/ZnO and transmittance spectrum of ZnO are presented in Figs. 1 and 2, respectively, which were utilized to investigate the mechanism of Fermi-level depinning effect at Al/ZnO/n-Ge interface. We conducted the XPS measurements for thick ZnO sample, ZnO/n-Ge interface sample, and pure Ge sample, to obtain the valence band offset (VBO) of ZnO/Ge, as shown in Fig. 1. The Zn 2*p* peak position and VBM for thick ZnO sample are 1021.9 eV and 2.59 eV, respectively. The Zn 2*p* and Ge 3*d* peak position for ZnO/Ge interface sample are 1021.7 eV and 29.1 eV, respectively. The Ge 3*d* peak position and VBM for pure Ge sample are 29 eV and 0.06 eV, respectively. This indicates that the VBO of ZnO/Ge is 2.33 eV [30].

Figure 2a shows the transmittance plot obtained from UV-VIS spectroscopy for thick ZnO sample, and the *E*_g_ of ZnO is determined to be 3.21 eV, consistent with the reported values in [28, 29]. By using the obtained *E*_g_ of ZnO and VBO above, a CBO of 0.22 eV between ZnO and Ge is determined, as shown in Fig. 2b. This indicates that Fermi-level depinning can be achieved at Al/n-Ge interface using the ZnO insertion layer, which can produce the low *R*_c_ for Al/ZnO/n-Ge contact.

**Fig. 3:**
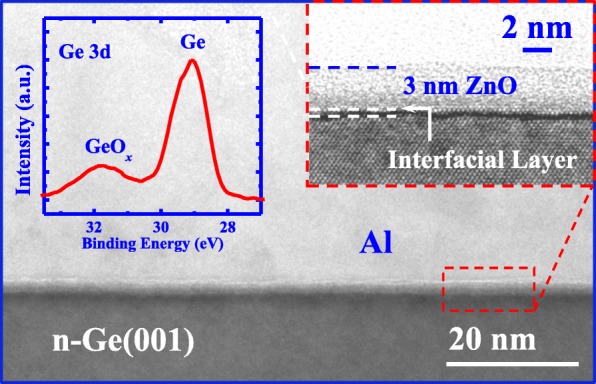
TEM image for an Al/ZnO/n-Ge sample showing the uniform ZnO layer between Al and Ge. Top right inset shows the HRTEM image of Al/ZnO/n-Ge interface, and the top left inset shows the XPS Ge 3*d* result of the sample demonstrating the existence of GeO_*x*_ interfacial layer

Figure 3 shows the TEM image of the Al/ZnO/n-Ge structure with the thickness of ZnO of 3 nm. A uniform and conformal ZnO layer is observed between Al and n-Ge. The inset in the top right corner illustrates the HRTEM image of zoomed-in view of the Al/ZnO/n-Ge interface. The thickness of ZnO film is measured to be 3 nm, which is consistent with result obtained by SE measurement, and the ZnO film is in an amorphous form.

A thin GeO_*x*_ interfacial layer is formed between Ge and ZnO, which is much smaller compared to [31] due to the lower deposition temperature used in this work. This is attributed to the fact that, during the deposition of ZnO, Ge reactive with O precursor, leading to the formation of GeO_*x*_ IL. GeO_*x*_ is also demonstrated by the XPS Ge 3*d* result in the inset in the top left corner.

**Fig. 4:**
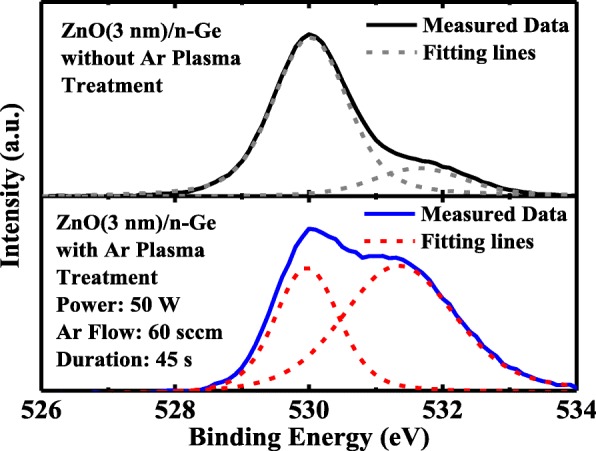
XPS results of O 1*s* and the deconvoluted results for as-deposited (upper) and Ar plasma-treated (lower) ZnO (3 nm)/n-Ge samples

Electrical conductivity of ZnO film can be improved by Ar plasma treatment, which causes the increasing in the concentration of oxygen vacancies *V*_o_, acting as the donors in ZnO [32, 33]. Figure 4 depicts the XPS results of O 1*s* for as-deposited ZnO and the sample with Ar plasma treatment with a power of 50 W, an Ar gas flow of 60 sccm, and a duration of 45 s. The O 1*s* peak is deconvoluted into two peaks by using the Gaussian fitting. The peak at ~ 530 eV corresponds to lattice oxygen in ZnO [34, 35]. For the as-deposited sample, the peak at 531.7 eV corresponds to *V*_o_ (~ 531.5 eV) and chemisorbed oxygen on the surface of ZnO thin films, such as carbonyl and hydroxyl groups [35, 37]. For the sample with Ar plasma treatment, the peak is at ~ 531.5 eV, which shifts to lower binding energy, and gets much more pronounced in comparison with the as-deposited sample, indicating that more *V*_o_ are generated due to Ar plasma treatment, and chemisorbed oxygen is effectively removed. The increasing of n-type dopants in ZnO results in the thinner tunneling barrier and lower series resistance at interface, being responsible for the reduction in *R*_c_ [36].

**Fig. 5:**
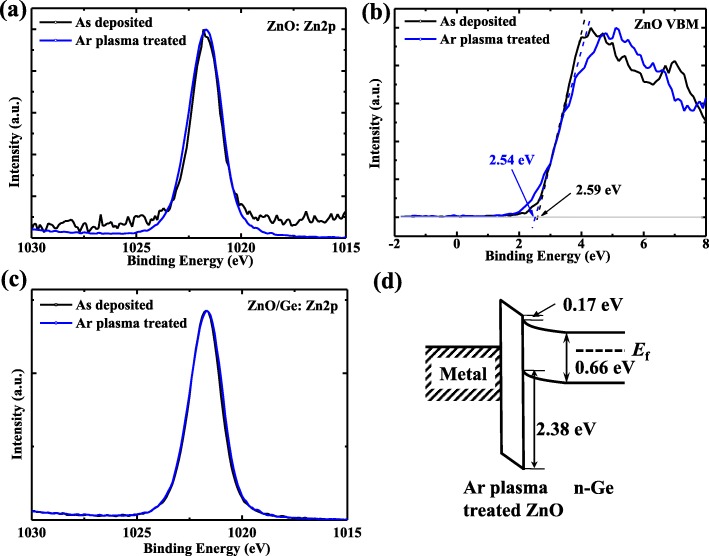
VBM for ZnO/Ge interface sample with and without Ar plasma treatment. **a** Zn 2*p* and **b** valence band spectra for thick ZnO sample **c** ZnO 2*p* spectra for ZnO/Ge interface sample. **d** Band alignment diagram for Ar plasma-treated metal/ZnO/n-Ge

We did the XPS measurements using thick ZnO sample and ZnO/Ge interface sample with and without Ar plasma treatment, as shown in Fig. 5. We found that, after Ar plasma treatment, there was a − 0.05 eV shift. This may indicate that the ZnO/Ge VBO is about 2.38 eV after Ar plasma treatment and CBO of 0.17 eV.

### Electrical Performance of Al/ZnO/n-Ge Contacts

**Fig. 6:**
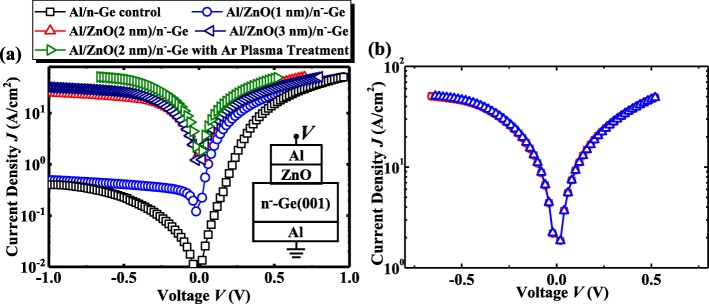
**a** *J*-*V* characteristics for Al/n^−^-Ge control, Al/ZnO/n^−^-Ge with ZnO thicknesses of 1 nm, 2 nm, and 3 nm, and Al/2 nm Ar plasma-treated ZnO/Ge, **b** *J*-V characteristics for three Al/2 nm Ar plasma-treated ZnO/Ge devices

Figure 6a shows the measured current density *J* as a function of applied voltage *V* characteristics for Al contacts on lightly doped n-Ge. The Al/ZnO/n^−^-Ge devices have the different thicknesses of ZnO layer. The schematic of the device is shown in the inset of Fig. 6.

As predicted, the Al/n-Ge control device without ZnO shows the rectifying characteristics with the high barrier height for electrons due to the Fermi-level pinning at Al/n^−^-Ge [38]. Compared with the control Al/n-Ge sample without ZnO, Al/ZnO/n-Ge devices exhibit the improved reverse *J*, which is due to the Fermi-level depinning induced by the reduction of metal-induced-gap-states (MIGS) at metal/Ge interface [18, 19]. This improvement is more enhanced with thicker ZnO, which is due to the fact that more MIGS are eliminated. But the forward current density for 3 nm ZnO inserted device is smaller than that of 2 nm one. This may be explained as follows. The main current density for Al/ZnO/n-Ge is tunneling current. If the ZnO is not thick enough, MIGS will not be effectively eliminated, and it still shows rectifying characteristics. But if the ZnO is too thick, the series resistance of ZnO will dominate the whole resistance, and the current gets smaller. So there is a trade-off between elimination of MIGS and increase in series resistance of ZnO, and thus there is a critical thickness for the IL [19]. In conclusion, 2 nm is considered to be the optimal thickness for Al/ZnO/n-Ge contact.

With the Ar plasma treatment, the performance of Al/ZnO/n^−^-Ge device is further improved. Whatever for the reverse or forward sweeping of applied voltage *V*, Al/2 nm ZnO/n^−^-Ge device with Ar plasma treatment achieves the enhanced *J* compared to the device with 2 nm ZnO or 3 nm ZnO, which is due to that a large amount of *V*_o_ are generated in ZnO film during the Ar plasma treatment. The higher doping concentration in ZnO can effectively reduce the series resistance of ZnO and reduce the tunneling barrier for electrons at the interface between ZnO and Al, improving the tunneling current density.

Figure 6b shows *J*-*V* characteristics for three Al/2 nm ZnO/n^−^-Ge device with Ar plasma treatment. It is clear that the *J* for different device is nearly the same, indicating that both ALD process and Ar plasma treatment have uniform effect on the improvement of current density.

**Fig. 7:**
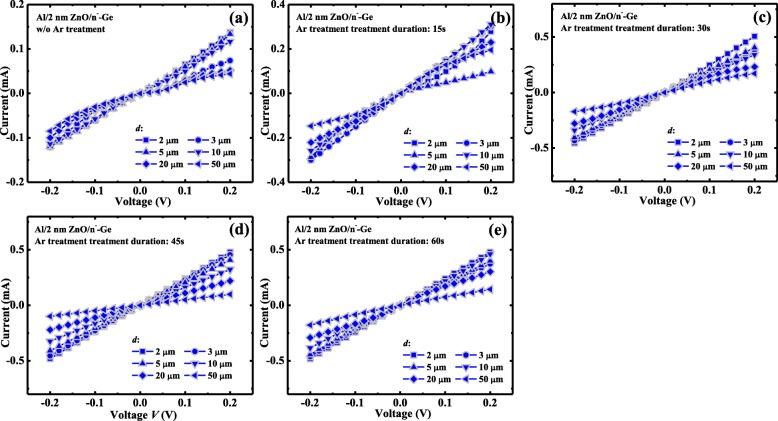
*I*–*V* curves at Al/2 nm ZnO/n^−^-Ge with different *d* **a** without Ar plasma treatment and with Ar plasma treatment duration of **b** 15 s, **c** 30 s, **d** 45 s, and **e** 60 s

Ohmic contacts are obtained for the Al/2 nm ZnO/n^−^-Ge without and with different Ar plasma treatment duration of 15 s, 30 s, 45 s, and 60 s, respectively, which are shown in Fig. 7.

**Fig. 8:**
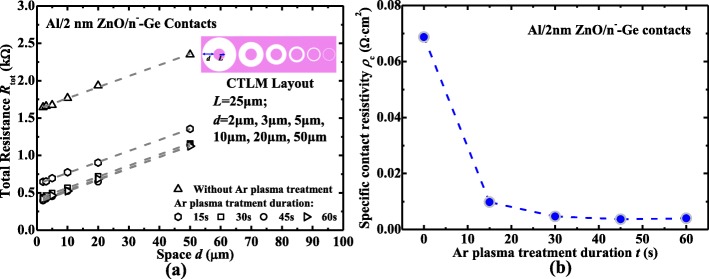
**a** *R*_tot_ versus *d* curves for the CTLM with Al/2 nm ZnO /n^−^-Ge contacts with different Ar plasma treatment duration, inset in Fig. 5a is the information of CTLM structure used in this work. **b** *ρ*_c_ versus different Ar plasma treatment duration

The raw total resistance *R*_tot_ between two contacts decreases with the decreasing of *d*, and the final *R*_tot_ is modified by a correction factor C, which is calculated with the equation *C* = (*L*/*d*)·ln(1 + *d*/*L*) [39], where *L* = 25 μm represents for the radius of inner pad, as depicted in the inset in Fig. 8a. By plotting the *R*_tot_ as a function of *d* in Fig. 8a, the sheet resistance *R*_sh_ of the n^−^-Ge can be obtained from the line slope, and then *ρ*_c_ is calculated from the intercept of the linear fitting line with the vertical axis. For the Al/2 nm ZnO/n^−^-Ge device without Ar plasma treatment, the *ρ*_c_ is 6.87 × 10^−2^ Ω cm^2^, but after 45 s Ar plasma treatment, there is 17.2 times reduction compared with that without Ar plasma treatment and has the contact resistivity *ρ*_c_ of 3.66 × 10^−3^ Ω cm^2^. We compare the values of *ρ*_c_ for the Al/2 nm ZnO/n^−^-Ge devices with different Ar plasma treatment durations in Fig. 8b. It is observed that *ρ*_c_ of the device decreases with the treatment time up to 30 s. However, as treatment time is larger than 30 s, *ρ*_c_ nearly stays the same. The reduction in *ρ*_c_ may be attributed to the doping of ZnO, thus to the reduction of tunneling barrier and series resistance, as has mentioned previously. But there is no observable change in sheet resistance of n^−^-Ge, indicating that there is no effect on the conductivity of n^−^-Ge with Ar plasma treatment.

**Fig. 9:**
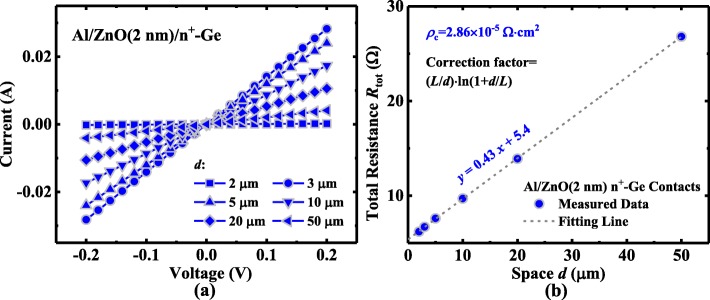
**a** *I*–*V* curves at Al/ZnO(2 nm)/n^+^-Ge with different *d* with ZnO treated using Ar plasma. **b** *R*_tot_ versus *d* curves for the CTLM with Al/ZnO(2 nm)/n^+^-Ge contacts

CTLM structure with Al contacts on heavily doped Ge is used to investigate the contact resistance characteristic of Al/2 nm ZnO/n^+^-Ge. The ZnO layer underwent the Ar plasma treatment for 45 s. Figure 9a shows the measured *I*-*V* curves between the Al contacts with different *d*, showing the excellent ohmic performance. Figure 9b plots the *R*_tot_ as a function of *d* for Al/2 nm ZnO/n^+^-Ge CTLM, and *R*_sh_ and *ρ*_c_ are extracted to be 64 Ω/□ and 2.86 × 10^−5^ Ω cm^2^, respectively.

**Fig. 10:**
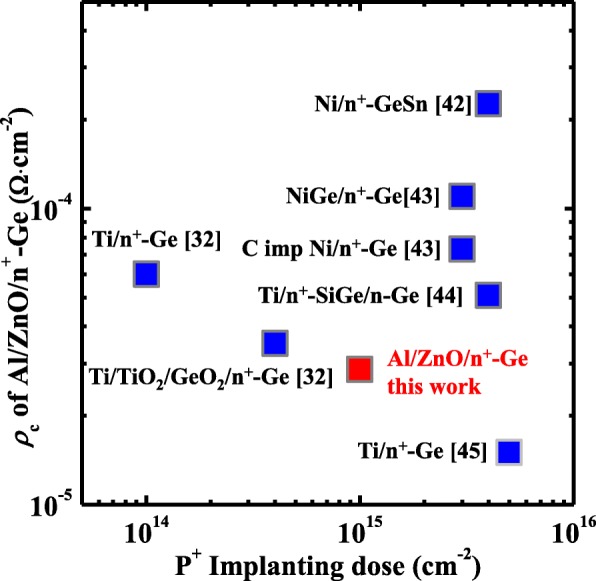
Comparison of *ρ*_c_ of Al/ZnO/n^+^-Ge in this work with those of other reported contacts, using P^+^ implantation dose as the *x* axis

We compare the *ρ*_c_ of ZnO treated by Ar plasma Al/ZnO/n^+^-Ge devices in this work with those reported in the literature, as shown in Fig. 10. For the heavily doped n^+^-Ge contact sample, Al/ZnO/n^+^-Ge contacts shown the smaller *ρ*_c_ in comparison with those of Ni/GeSn [40, 41], Ni/Ge [42], Ti/n^+^-Ge in ref. [31], and Ti/TiO_2_/GeO_2_/Ge [31], carbon implanted Ni/Ge [42], and Ti/n^+^-SiGe/n-Ge [43]. Metallic ohmic contacts such as Ni/Ge, Ni/GeSn, Ti/Ge, and carbon implanted Ni/Ge suffer from severe Fermi-level pinning, resulting in the large *ρ*_c_. For Ti/TiO_2_/GeO_2_/Ge contact, a large tunneling resistance was introduced by the bilayer of 1 nm TiO_2_/1.5 nm GeO_2_ IL, degrading the contact resistivity characteristics. But the *ρ*_c_ in this work is larger than that in ref. [44]. We assume that this may due to the four times larger P^+^ implantation dose than that in our work. Larger implantation dose will enable the heavier surface doping of n^+^-Ge, resulting in the thinner Schottky barrier and smaller *ρ*_c_. We believe that with heavier doping of n^+^-Ge in Al/ZnO/n^+^-Ge devices, smaller *ρ*_c_ will result in.

## Conclusions

The Fermi-level depinning effect induced by ZnO IL in the Al/ZnO/n-Ge structures is investigated. XPS measurement demonstrated a small CBO of 0.22 eV at ZnO/n-Ge, i.e., elimination of FLP occurs, which leads to the ohmic metal contacts on n-Ge. It is further reported that Ar plasma treatment of ZnO leads to the increasing of concentration of *V*_o_, acting as the n-type dopants in ZnO, which improves the *R*_c_ performance in Al/ZnO/n-Ge devices. Ohmic metal contacts are obtained on n^−^ and n^+^-Ge with the Ar plasma-treated ZnO IL. Based on the CTLM structures, values of *ρ*_c_ 3.66 × 10^−3^ Ω cm^2^ and 2.86 × 10^− 5^ Ω cm^2^ are achieved in Al/2 nm ZnO/n^−^-Ge and Al/2 nm ZnO/n^+^-Ge, respectively, with the Ar plasma treatment of ZnO at a power of 50 W for 45 s.

## Abbreviations

Al: Aluminum
ALD: Atomic layer deposition
Ar: Argon
CBO: Conduction band offset
CTLM: Circular transmission line method
DEZn: Diethyl zinc
*E*_g_: Band gap
FLP: Fermi-level pinning
Ge: Germanium
GeO_*x*_: Germanium oxide
GeSn: Germanium tin
HCl: Hydrochloric acid
HRTEM: High-resolution transmission electron microscope
IL: Interfacial layer
MIGS: Metal-induced-gap-states
MOSFETs: Metal-oxide-semiconductor field-effect transistors
Ni: Nickel
P^+^: Phosphor ion
*R*_c_: Contact resistance
*R*_tot_: Raw total resistance
SE: Spectroscopic ellipsometry
Si: Silicon
Ti: Titanium
TiO_2_: Titanium dioxide
UV-VIS: Ultraviolet–visible
VBO: Valence band offset
*V*_o_: Oxygen vacancy
XPS: X-ray photoelectron spectroscopy
ZnO: Zinc oxide
*ρ*_c_: Specific contact resistivity
